# Prenatal Features of MIRAGE Syndrome—Case Report and Review of the Literature

**DOI:** 10.3390/children11030310

**Published:** 2024-03-05

**Authors:** Anca Maria Panaitescu, Iulia Huluță, Gabriel-Petre Gorecki, Luminita Nicoleta Cima, Vlad M. Voiculescu, Florina Mihaela Nedelea, Nicolae Gică

**Affiliations:** 1 Department of Obstetrics and Gynecology, Faculty of Medicine, Carol Davila University of Medicine and Pharmacy, 020021 Bucharest, Romania; iulia.huluta@drd.umfcd.ro (I.H.); luminita.cima@umfcd.ro (L.N.C.); vlad.voiculescu@umfcd.ro (V.M.V.); gica.nicolae@umfcd.ro (N.G.); 2 Department of Obstetrics and Gynecology, Filantropia Clinical Hospital Bucharest, 011171 Bucharest, Romania; genetica@spitalulfilantropia.ro; 3 Faculty of Medicine, Titu Maiorescu University, 031593 Bucharest, Romania; gabriel.gorecki@prof.utm.ro; 4 Department of Anesthesia and Intensive Care, CF2 Clinical Hospital, 011464 Bucharest, Romania; 5 Department of Endocrinology and Diabetes, Nutrition and Metabolic Diseases, Elias Emergency University Hospital, 011461 Bucharest, Romania; 6 Department of Dermatology, Carol Davila University of Medicine and Pharmacy, 050474 Bucharest, Romania

**Keywords:** MIRAGE syndrome, prenatal diagnosis, fetal growth restriction, SAMD9, WES

## Abstract

MIRAGE syndrome is a recently described congenital condition characterized genetically by heterozygous gain-of-function missense mutations in the growth repressor sterile alpha domain containing 9 (SAMD9) located on the arm of chromosome 7 (7q21.2). The syndrome is rare and is usually diagnosed in newborns and children with myelodysplasia, infection, restriction of growth, adrenal hypoplasia, genital phenotypes, and enteropathy, hence the acronym MIRAGE. The aims of this paper are (1) to present fetal ultrasound features in a case where MIRAGE syndrome was diagnosed prenatally and (2) to review the existing literature records on prenatal manifestations of MIRAGE syndrome. In our case, the fetus had severe early fetal growth restriction (FGR) with normal Doppler studies, atypical genitalia, oligohydramnios, and hyperechogenic bowel at the routine mid-gestation anomaly scan. Amniocentesis excluded infections and numeric or structural chromosomal abnormalities while whole exome sequencing (WES) of the fetal genetic material identified the specific mutation. Targeted testing in parents was negative, suggesting the “de novo” mutation in the fetus. We could not identify other specific case reports in the literature on the prenatal diagnosis of MIRAGE syndrome. In cases reported in the literature where the diagnosis of MIRAGE syndrome was achieved postnatally, there are mentions related to the marked FGR on prenatal ultrasound. Severe early-onset FGR with no other apparent cause seems to be a central prenatal feature in these babies, and WES should be offered, especially if there are other structural abnormalities. Prenatal diagnosis of MIRAGE syndrome is possible, allowing for reproductive choices, improved counseling of parents, and better preparation of neonatal care.

## 1. Introduction

MIRAGE syndrome is a recently recognized condition, first described in 2016. This rare genetic syndrome is characterized by multisystem growth restriction in association with primary adrenal hypoplasia, and it is usually diagnosed in early life. The syndrome’s name is an acronym of the first letters of its main features: myelodysplasia, infection, restriction of growth, adrenal hypoplasia, genital phenotypes, and enteropathy. Babies with MIRAGE syndrome are usually born preterm, with significant growth restriction [1]. Thrombocytopenia, anemia or pancytopenia are typically diagnosed soon after birth, and early infancy is complicated by recurrent infections (bacterial, viral, or fungal) and growth restriction. Disorders of sexual development are common and genital phenotypes in 46, XY children vary from hypospadias, microphallus, and atypical/ambiguous genitalia to complete female genitalia. Gastrointestinal issues include chronic diarrhea, severe enteropathy, esophageal reflux, aspiration, and need for tube feeding. There may be salt-losing primary adrenal insufficiency. Many of the affected babies die and those who survive have moderate-to-severe developmental delay [2]. MIRAGE syndrome is usually diagnosed in a child with suggestive features when genetic testing, whole exome sequencing (WES), reveals a heterozygous gain-of-function missense mutations in the growth repressor sterile alpha domain containing 9 (SAMD9) located on the arm of chromosome 7 (7q21.2) [3,4]. 

Prenatal ultrasound is now offered to all pregnant women and allows visualization of the developing fetus and the early detection of fetal abnormalities. When ultrasound findings raise suspicions of genetic disorders, invasive prenatal diagnostic procedures such as amniocentesis may be performed to obtain fetal cell samples which can then be analyzed for chromosomal abnormalities (by karyotyping and chromosomal microarray analysis, CMA) and genetic mutations (targeted or whole genome, next-generation sequencing (NGS), or whole exome sequencing, WES). These tests are increasingly used prenatally and allow accurate diagnosis of genetic conditions even before birth and facilitate adequate preparation and counseling of the parents.

The focus of this paper was directed towards two key aims:

(1) To present prenatal features in a case of MIRAGE syndrome diagnosed prenatally in a fetus with severe growth restriction, ambiguous genitalia, hyperechogenic bowel, and oligohydramnios at the mid-gestation routine anomaly scan, at 22 weeks of pregnancy.
(2) To review the literature in search of reports on prenatal features of MIRAGE syndrome.

## 2. Materials and Methods

In conducting this review of the existing literature, we searched on the 20th of January 2024 PubMed, Web of Science and Google Scholar for the term “Mirage syndrome”. The search revealed 46 entries in PubMed, 77 in Web of Science, and 155 in Google Scholar. To refine our selection process and ensure the relevance of the gathered literature to our study objectives, we then selected the published articles that contained case reports, and, from those, we excluded duplicates and only included for this study those articles in the English language with reference to prenatal features. We did not include meeting abstracts. The main focus of this research was to understand prenatal phenotypes of MIRAGE syndrome; therefore, the focus of our search was on these aspects. Increasing awareness of prenatal manifestations of MIRAGE syndrome would familiarize clinicians involved in fetal ultrasound recognize this syndrome and offer adequate genetic testing to willing parents. We also present our own case of MIRAGE syndrome diagnosed prenatally.

## 3. Results

### 3.1. Case Presentation

**Figure 1:**
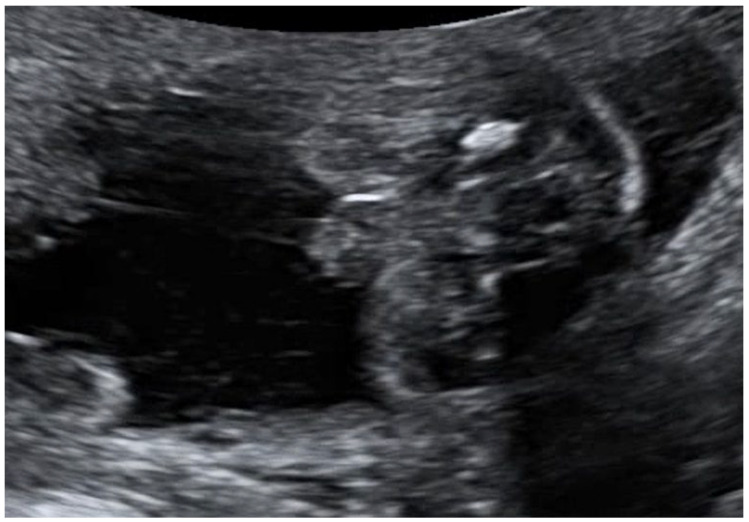
Ultrasound image of ambiguous genitalia characterized by the ‘tulip sign’, observed in a 46, XY fetus during the 20th week of gestation.

We present a case of a 30-year-old primigravida, with no relevant medical history who had an uneventful pregnancy up to the point of her routine second-trimester ultrasound anomaly scan, at 20 weeks’ gestation. In our service, all pregnant women are offered three detailed fetal scans throughout pregnancy: first at 11 to 13 weeks for dating, estimating risks of common chromosomal abnormalities, structural check-up, and preeclampsia screening; the second is planned around 20–24 weeks for fetal anomaly scan and cervical length measurement, and the third is at 30–34 weeks to check for growth and fetal dopplers [5,6]. In the presented case, the risk for common chromosomal abnormalities estimated by the first trimester combined scan was low. Common blood tests were normal and ruled out maternal infections. There was no consanguinity. Dating of pregnancy was performed as per guidelines by first-trimester measuring of crown-to-rump length [7]. At the second-trimester anomaly scan, the fetus was measured small-for-gestational age, with a 2-week growth delay, there were oligohydramnios (deepest amniotic fluid pool of 2 cm), ambiguous genitalia (Figure 1), and hyperechogenic bowel [8]. Doppler measurements in the umbilical artery, ductus venosus, middle cerebral artery, and uterine arteries were normal, making the diagnosis of fetal growth restriction due to placental insufficiency less likely [9]. There were no features of fetal infections or other structural anomalies. The parents were informed and consented to invasive prenatal genetic testing by amniocentesis. Amniotic fluid was sent for initial genetic testing (karyotype, SNP array, a gene panel for cystic fibrosis because of the hyperechogenic bowel) and fetal infections [10]. Fetal karyotype indicated a normal male fetus, 46, XY, and the SNP array came back normal. Fetal infections (Cytomegalovirus, Toxoplasma gondii) and cystic fibrosis were ruled out. After the initial normal results, the parents were informed about the possible monogenic etiology of the fetal features seen on the scan and the next available options of testing—whole exome Sequencing (WES) and whole genome sequencing and opted for this test. We do store, per protocol in our unit, with patients’ consent, the amniotic fluid after the initial invasive procedure for future testing if required. Parents agreed to WES which identified a heterozygous variant, of uncertain significance, NM_001193307.1:c.2054G>A p.(Arg685Gln), in the SAMD9 gene, making the diagnosis of autosomal dominant MIRAGE syndrome possible. This missense variant, c.2054G>A p.(Arg685Gln), that causes an amino acid change from Arg to Gln at position 685, has previously been described as disease-causing for MIRAGE syndrome by Buonocore et al. [4]. Further, parental testing was undertaken, and the fetal variant was not identified in parental tests, demonstrating the “de novo” origin in the fetus. Therefore, because the fetal variant is absent in healthy cohorts and is missing in familial segregation, due to the availability of this evidence, the variant has been reclassified from a variant of uncertain significance (class 3) to likely pathogenic (class 2), confirming the diagnosis of MIRAGE syndrome in the fetus. After extensive counseling, the parents opted for termination of pregnancy. Counseling included a discussion on the risk of future pregnancies, which was estimated to be low because the variant identified in the index was de novo (although, rarely germline mosaicism in one of the parents is possible). Despite the challenges posed by the COVID-19 pandemic, the clinical management and work-up for this case remained unhindered. The dedication of healthcare professionals and the implementation of safety protocols ensured the timely retrieval of results and the provision of necessary care for the parents [11].

### 3.2. Literature Review

MIRAGE syndrome has recently been described and recognized as a specific syndrome in neonates and children. In 2016, Narumi et al., reported on heterozygous gain-of-function mutations in SAMD9 in 11 patients with growth restriction apparent from fetal life, adrenal insufficiency, and gonadal failure, together with bone marrow failure [3]. In 2017, Buonocore et al., reported SAMD9 mutations using NGS techniques in eight patients with multisystem growth restriction phenotypes. The authors showed that complex dynamic somatic changes in SAMD9 and the SAMD9/SAMD9L locus on chromosome 7q are associated with the distinct MIRAGE syndrome phenotype and modify survival in affected patients [12]. Sterile α motif domain containing protein 9 (SAMD9, OMIM 610456) is a 1589–amino acid protein that is encoded by a gene on the long arm of chromosome 7 (7q21.2). SAMD9 is likely to act as a growth suppressor [13]. Mortality is high and children who survive show long-term growth restriction. As many of these features are found in sick, preterm, growth-restricted babies, this condition is likely underdiagnosed.

A recent systematic review by Suntharalingham et al., found 116 cases of MIRAGE syndrome reported in the literature all diagnosed postnatally. With our search, we found only nine case reports of postnatally diagnosed MIRAGE syndrome where there is a reference to prenatal features on ultrasound in the affected patients (Table 1). The main feature in the prenatal period seems to be severe early-onset FGR. Most of the affected babies had an iatrogenic preterm birth, by caesarean section for fetal distress [14]. Other features of MIRAGE syndrome that can be recognized prenatally are atypical genitalia and anomalies of the reno–urinary system. 

Given the ubiquity of prenatal ultrasound technologies and the increased accessibility of invasive testing, which includes advanced methodologies like next-generation sequencing (NGS) and whole exome sequencing (WES) for genetic material analysis, the diagnosis of MIRAGE syndrome has now become feasible during the prenatal period. These advancements in diagnostic tools empower healthcare professionals to identify and characterize MIRAGE syndrome in utero, enabling early recognition and potential intervention.

## 4. Discussion

MIRAGE syndrome is a recently described congenital condition characterized genetically by heterozygous gain-of-function missense mutations in the growth repressor sterile alpha domain containing 9 (SAMD9) located on the arm of chromosome 7 (7q21.2). Sterile α motif domain–containing protein 9 (SAMD9, OMIM 610456) is a 1589–amino acid protein that is encoded by a gene on the long arm of chromosome 7 (7q21.2). SAMD9 is likely to act as a growth suppressor [3]. Mortality is high and children who survive show long-term growth restriction. As many of these features are found in sick, preterm, growth-restricted babies, this condition is likely underdiagnosed. We aimed to describe the prenatal features of MIRAGE syndrome and increase awareness among medical providers involved in pregnancy care about this syndrome. The main feature is fetal growth restriction; however, an attentive ultrasound fetal scan can reveal some other additional minor features that could suggest the diagnosis and prompt clinicians to offer invasive testing. As with all prenatal genetic diagnoses, the problem of predicting the postnatal phenotype remains. Understanding the spectrum of presentations of MIRAGE syndrome aids in refining genetic counseling and offering more informed reproductive choices to parents; involving a senior geneticist in the process is crucial.

**Figure 2:**
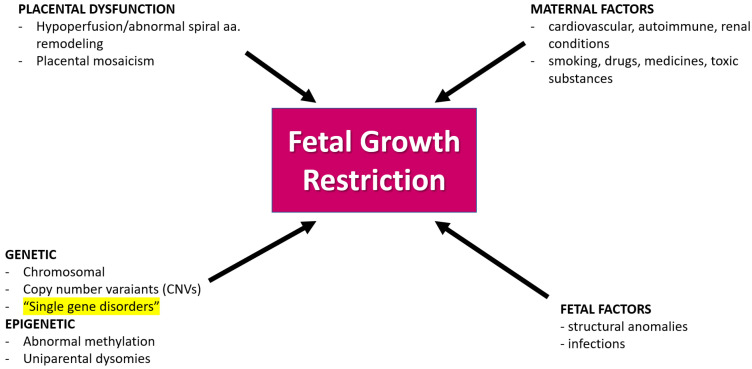
Causes of fetal growth restriction in prenatal medicine. Most frequently, FGR is a consequence of placental insufficiency. Other causes are maternal disorders, maternal exposure to drugs or toxic factors, and fetal factors like infections or structural abnormalities. Single gene disorders, like in the case we present here, are rare causes of FGR (highlighted in Figure 2), nevertheless, they should be taken into consideration in the differential diagnosis; aa—arteries.

In the case we present, the suspicion of a fetal genetic abnormality was raised at the mid-trimester anomaly scan in a baby with severe growth restriction with normal Doppler studies, negative tests for infections, ambiguous genitalia, hyperechogenic bowel, and oligohydramnios. Differential diagnosis of the fetal condition was an important part of solving this case (Figure 2). Both fetal and maternal aspects should be considered when making a diagnosis in fetal medicine. Within the realm of maternal health, there exists a spectrum of conditions, each with its unique implications for fetal development. Notably, some maternal conditions like hypertension, diabetes and endocrinopathies, neurological or autoimmune disease possess the ability to potentially increase the risk of adverse pregnancy outcomes. Moreover, maternal medication, environmental factors, and drugs can have teratogenic effects on the developing fetus. The intricate interplay between maternal health and fetal well-being is a subject of extensive research and clinical consideration. In our case, we excluded any maternal condition or exposure that can be linked to the fetal congenital defects seen [23].

Fetal growth restriction stands as a frequently encountered diagnosis within the field of fetal medicine, representing a complex scenario where the developing fetus fails to achieve its expected genetic potential in terms of growth. This condition poses a considerable challenge for healthcare providers, necessitating a comprehensive understanding of its diverse etiological factors and potential implications for both the fetus and the expecting mother. The primary driver behind FGR is often identified as placental insufficiency, a condition where the placenta, a vital organ facilitating nutrient and oxygen exchange between the mother and the fetus, falls short of meeting the developmental demands of the growing baby. In early FGR, due to placenta insufficiency, doppler studies in uterine arteries, in the umbilical artery, and the ductus venosus are often abnormal; therefore, in experienced hands, the diagnosis of FGR of placenta origin is often easy. These cases are not usually associated with chromosomal and genetic causes. However, it is crucial to acknowledge that FGR can manifest through a multitude of contributing factors, further emphasizing the intricacy of its origins. In addition to placental insufficiency, a spectrum of other potential culprits can contribute to FGR. Infections, whether viral or bacterial, pose a risk by disrupting the delicate balance required for optimal fetal growth. In cases where FGR is caused by fetal infections, we can see other signs of fetal involvement as well such as calcifications and involvement of the brain or liver. Exposure to certain toxins and medications during pregnancy can also play a role, underscoring the importance of carefully considering maternal environmental factors in the context of fetal development. Maternal conditions add another layer of complexity to the landscape of FGR. Underlying health issues, such as hypertension, diabetes, or autoimmune disorders, can impact the maternal–fetal interface, potentially influencing the growth trajectory of the developing fetus. The intricate interplay of these various factors requires a nuanced and multidisciplinary approach for accurate diagnosis and effective management. Furthermore, it is essential to recognize the potential involvement of concomitant fetal structural anomalies in cases of FGR. The coexistence of these structural issues with growth restriction adds layers of complexity to the clinical picture, demanding a thorough evaluation to guide appropriate interventions and counseling for expecting parents. In a subset of cases, particularly when FGR manifests early in pregnancy, often before 32 weeks, and is associated with minor ultrasonographic findings, like in the case we present here, genetic causes and syndromes come into focus. Unraveling the genetic underpinnings of FGR is a challenging yet crucial aspect of fetal medicine, contributing not only to diagnostic precision but also shaping the trajectory of genetic counseling and potential interventions [24]. When FGR is suspected antenatally, genetic invasive testing is taken into consideration. Traditionally, karyotype and CMA have been employed; however, recent studies have proven that newer technologies like NGS improve the diagnostic yield. In a recent systematic review and metanalysis, WES resulted in a 12% (95% CI: 7–18%) incremental performance over that achieved by chromosomal microarray analysis (CMA) or karyotyping in fetuses with isolated FGR, the vast majority before 32 weeks of pregnancy [25]. The study included eight studies with 146 fetuses with isolated FGR. The aim of the study was to determine the diagnostic yield of WES above that of chromosomal microarray or karyotyping in fetuses with isolated growth restriction. Selected studies included fetuses with FGR in the absence of major fetal structural anomalies with negative CMA and karyotyping results. The results of WES were considered causative when only positive variants classified as likely pathogenic or pathogenic were determined. In this study, a pathogenic variant determined as causative of the fetal phenotype was found in 17 cases out of the 146 fetuses with isolated FGR before 32 weeks of pregnancy [25]. 

In our case, FGR was associated with hyperechogenic bowel, oligohydramnios, and ambiguous genitalia. Hyperechogenic bowel is a subjective assessment of the echogenicity of the fetal bowel on ultrasound and is another common finding in prenatal medicine. It may be a sign of intrauterine enteropathy as it probably is in the case presented here; however, it is a non-specific sign, apparent in other many fetal conditions [26]. In fetuses with FGR of placental insufficiency, hyperechogenic bowel is a marker of mesenteric ischemia. Hyperechogenic bowel can also be seen in infections such as cytomegalovirus infection and cystic fibrosis and as a transient finding after intra-amniotic bleeding. Oligohydramnios may be a sign of renal impairment in the case we present, however, it presents itself in many other fetal conditions as well [27]. In typical FGR of placenta ischemia, oligohydramnios shows inadequate fetal renal perfusion. Oligohydramnios can be a consequence of abnormal fetal kidney function, obstructive uropathies, ruptured membranes, and infections, and it can be found in many other disease-related scenarios. 

Atypical genitalia are a key feature seen postnatally in newborns with MIRAGE syndrome. Atypical genitalia can, in many cases, be seen from fetal life in the second or third trimester. In a study by van Bever at al., atypical genitalia were correctly diagnosed from fetal life in 91% of fetuses with this isolated finding [28]. Sexual development is coordinated by specific genes that interplay to differentiate the bipotential gonads of a growing fetus into either ovaries or testis followed by the differentiation of external and internal genital systems after exposure to specific hormones. Differences in sex development (DSD) happen when there are congenital alterations during the complex process of genital organ development and are classified as 46,XY DSD when the genetic sex chromosome found is Y and 46,XX DSD when the genetic sex chromosome is X. For proper management and counseling, understanding the genetics and the embryology of typical and atypical genital organ development is required. Recent insights have been gained in understanding the genetic background of DSD, especially 46,XY DSD with the use of new tools like WES having been more and more introduced in clinical practice. A growing body of research is focused on understanding the future genes involved in typical and atypical sex development and in improving our understanding of DSD [29]. 

Prenatally, atypical genitalia are notoriously associated with genetic anomalies in the fetus, and it is a situation where counseling parents is very challenging [28,30]. The association of FGR with atypical genitalia should trigger investigations into fetal genetic syndromes, as it has done in our case. In a study of Leitao Braga et al. that included 46 individuals with hypospadias from a large cohort of 46,XY DSD patients, there were 5 individuals with specific genetic syndromes: 3 with mutations specific to Silver-Russell syndrome and 2 de novo pathogenic variants in a compound heterozygous state were identified in the CUL7 gene, establishing the diagnosis of 3M syndrome in one patient, and a novel homozygous variant in TRIM37 was identified in another boy with Mulibrey nanism phenotype [31]. 

In the medical literature, fetal growth restriction emerges as a primary prenatal characteristic of MIRAGE syndrome. Infants subsequently diagnosed with MIRAGE syndrome are often delivered preterm through iatrogenic means, prompted by concerns of fetal distress, maybe related to adrenal insufficiency.

MIRAGE syndrome, stemming from pathogenic SAMD9 variants, manifests as a congenital multisystem disorder with features like 46, XY differences in sex development (DSD), small for gestational age (SGA), and adrenal insufficiency (AI). Despite the commonality of AI in MIRAGE, Narumi et al., identified a 46, XY DSD SGA case without AI, broadening the spectrum of this rare syndrome. The patient harbored a novel SAMD9 variant and exhibited diverse MIRAGE-associated traits, highlighting the need for comprehensive evaluation beyond AI for an accurate diagnosis [32].

Expanding the understanding of MIRAGE syndrome caused by heterozygous de novo SAMD9 variants, Buoncore et al., delved into its phenotypic variability. Notably, MIRAGE features were observed without adrenal dysfunction in certain cases, emphasizing the evolving clinical landscape. The presence of endocrine disease, including hypospadias, and the absence of specific SAMD9 variants in pregnancy loss and growth restriction cohorts underscore the syndrome’s complexity and potential underdiagnosis. Precise diagnosis becomes pivotal for tailored management in the diverse clinical scenarios associated with MIRAGE syndrome [12].

Another paper presented two patients with MIRAGE syndrome that exhibited activating SAMD9 mutations along with second-site reversion nonsense mutations on the same allele. Patient 1 had p.Arg685 identified as a somatic mutation, suggesting a potential reversal of their growth restriction. Patient 2, with p.Gln39, showed skewed X chromosome inactivation, indicating monoclonality. These cases represent the first instances of complete hematological reversion in MIRAGE syndrome, potentially mitigating thrombocytopenia and anemia. Patient 2 displayed dysautonomic symptoms, while catch-up growth and normal postnatal growth in some patients suggest variability in the syndrome’s manifestations. Acquisition of somatic nonsense SAMD9 mutation in the cells of the hematopoietic system might revert the cellular growth repression caused by the germline SAMD9 mutations. The unexpected lack of hematological features in the two patients would be explained by the reversion mutations [33,34,35]. Further studies are needed for conclusive insights. 

## 5. Conclusions

In summary, our study brings a spotlight to the prenatal diagnosis of MIRAGE syndrome, with a particular focus on utilizing the identification of severe fetal growth restriction (FGR) as a pivotal starting point. Our case, uncovered during routine mid-gestation anomaly scanning, not only reveals the intricacies of this rare genetic condition, including atypical genitalia, hyperechogenic bowel, and oligohydramnios, but also highlights the significance of recognizing FGR as a key indicator. Through a review of the existing literature, we wanted to expand our understanding of the prenatal features associated with MIRAGE syndrome. Significantly, our findings underscore the crucial role played by prenatal tools like ultrasound and genetic testing, especially whole exome sequencing, in enabling early diagnosis and empowering parents to make informed decisions about reproduction. Our research amplifies the call for heightened awareness, prompt diagnosis, and supportive counseling for parents, all of which collectively contribute to enhanced neonatal care. Moreover, we consider that future research should aim at unraveling the diverse phenotypic expressions of MIRAGE syndrome, starting with the early identification of FGR and the associated phenotypic prenatal features. Raising awareness of this condition and having greater access to rapid genetic sequencing could be essential to identify children who harbor pathogenic SAMD9 variants, and to identify some of the “missing” cohorts of 46, XX girls with this condition. Indeed, more widespread use of whole exome/genome sequencing for fetuses and children with growth restriction and associated features is likely to identify more children with MIRAGE syndrome and provide more information on the range of phenotypic features.

## Figures and Tables

**Table 1:** Gestational and postnatal features in MIRAGE syndrome: insights for prenatal diagnosis [14,15,16,17,18,19,20,21,22]; CS—caesarean section; GA—gestational age; FGR—fetal growth restriction.

Author, Year	GA at Delivery, Indication	Prenatal Features	Postnatal Features
Go et al., 2023 [14]	29 weeks + 6 days Iatrogenic—CS for fetal distress and oligohydramnios	FGR Lung hypoplasia Pericardial effusion Bilateral renal hypoplasia	Hyperpigmentation Adrenal insufficiency Dysplastic Kidneys Normal genitalia Transient thrombocytopenia Severe developmental delay at 5–6 months
Onuma et al., 2020 [15]	31 weeks Iatrogenic—CS for fetal distress	FGR	Micropenis Hypospadias Bifid scrotum Hyperpigmentation Adrenal insufficiency Bowel dysfunction Transient thrombocytopenia
Yoshizaki et al., 2019 [16]	32 weeks + 2 days Iatrogenic—CS fetal distress	FGR	Hyperpigmentation Adrenal insufficiency Transient thrombocytopenia Normal genitalia
Janjua et al., 2022 [17]	34 weeks + 5 days Iatrogenic—CS	FGR Right hydroureter Female external genitalia NIPT male genotype.	Prominent clitoris Small vaginal opening Small lumps in the groin No fusion of the labia Normal appearance of the lower vagina ending blindly with no visible cervix Normal female urethra No ovaries
Roucher-Boulez et al., 2019 [18]	36 weeks + 5 days Iatrogenic—CS for fetal distress	FGR	Very small, inguinal palpable testesUrogenital sinus Blind-ending vagina Genital tubercle had the appearance of a normal clitoris The karyotype was 46, XY Transient thrombocytopenia
Mengen et al., 2020 [19]	31 weeks Iatrogenic—CS for fetal distress and FGR	FGR	Micropenis Transient thrombocytopenia Adrenal insufficiency due to bilateral adrenal hypoplasia Bowel dysfunction
Baquedano-Lobera et al., 2021 [20]	31 weeks	FGR	
Zhang et al., 2019 [21]	31 weeks	FGR	Hyperpigmentation Dysmorphic features Hypoplastic genitalia Visible penis, no visible testis and scrotum. Left testicle in the inguinal canal, right testicle in the right lower pelvic cavity Undetected bilateral epididymis No solid mass found in bilateral scrotum
Perisa et al. (2019) [22]	32 weeks CS—Oligohydramnios and FGR	FGR	Microcephaly Cryptorchidism Hypospadias Unilateral vesicoureteral reflux Mild facial dysmorphism Brachydactyly of bilateral fifth digits

## Data Availability

Not applicable.
